# Risk-centered benchmarking of large language models for AI-enabled counseling in chronic autoimmune thyroid eye disease

**DOI:** 10.3389/fcell.2026.1871740

**Published:** 2026-06-15

**Authors:** Fangqin Fei, Lu Xie, Jing Rao, Ziqi Liang, Juan Yang, Yunyun Zou, Ligang Jiang

**Affiliations:** 1 Department of Endocrinology, The First People’s Hospital of Huzhou, Huzhou Normal University, Huzhou, Zhejiang, China; 2 Shenzhen Eye Hospital, Shenzhen Eye Medical Center, Southern Medical University, Shenzhen, Guangdong, China; 3 Department of Ophthalmology, Quzhou People’s Hospital, The Quzhou Affiliated Hospital, Wenzhou Medical University, Wenzhou, China

**Keywords:** artificial intelligence, autoimmune inflammation, chronic ocular disease, large language models, risk stratification, thyroid eye disease

## Abstract

**Background:**

Thyroid eye disease (TED) is a chronic autoimmune inflammatory orbital disease requiring activity assessment, risk stratification, and triage. As patients increasingly consult large language models (LLMs), evidence on their quality and safety for TED counseling remains limited.

**Methods:**

We conducted a cross-sectional benchmark using a prespecified 35-question Chinese TED counseling bank covering symptom recognition, activity assessment, treatment, daily management, follow-up, and care-seeking. The bank was developed from guideline-/consensus-derived scenarios, expert discussion, and recurrent patient-inquiry themes, then applied under a unified single-turn protocol. Five web-based LLM chatbots were evaluated: Gemini 3 Pro, ChatGPT-5.2, DeepSeek-V3.1, Doubao, and Qwen3-Max. Systems were accessed through official interfaces in Quzhou, China, during 27–29 December 2025, with identifiers recorded. Automated text analysis extracted output features, and response time was measured. Two blinded expert raters assessed alignment with a guideline-/consensus-informed reference standard using 5-point Likert scales for Accuracy, Logic, Coherence, Safety, and Content Accessibility. Between-model comparisons used repeated-measures methods, and correlations used Spearman analysis.

**Results:**

Response time differed significantly across models (Friedman χ^2^ = 94.79, P < 0.001), with Gemini 3 Pro fastest and Doubao slowest. Output characteristics varied: Doubao generated the longest responses, ChatGPT-5.2 the shortest, and Qwen3-Max the most table-formatted outputs. Significant between-model differences were found for Accuracy (χ^2^ = 16.64, P = 0.002), Logic (χ^2^ = 20.76, P < 0.001), Coherence (χ^2^ = 15.54, P = 0.004), and Content Accessibility (χ^2^ = 23.51, P < 0.001), but not Safety (χ^2^ = 1.03, P = 0.905). Response time correlated moderately with output length (words ρ = 0.53; characters ρ = 0.51). Content Accessibility correlated weakly with length and table use (tables ρ = 0.29), whereas longer outputs were not consistently associated with higher Accuracy or Logic.

**Conclusion:**

LLMs show marked heterogeneity in efficiency, output structure, and clinical quality for TED counseling. Longer or slower responses do not necessarily indicate better performance. The absence of between-model Safety differences should not be interpreted as absolute safety or equivalence. Risk-centered, structured outputs emphasizing red-flag symptoms and care-seeking thresholds warrant validation through multi-turn dialogues, repeated sampling, patient/lay-user evaluation, and finer-grained safety endpoints.

## 1 Introduction

**FIGURE 1:**
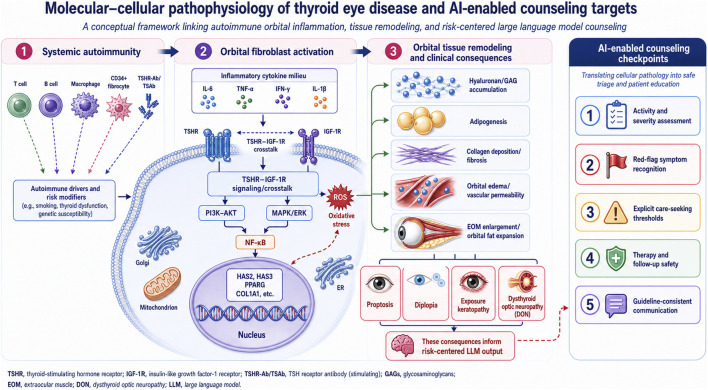
Molecular–cellular pathophysiology of thyroid eye disease and artificial intelligence (AI)-enabled counseling targets. Thyroid eye disease (TED) involves systemic autoimmunity, inflammatory cytokine signaling, thyroid-stimulating hormone receptor (TSHR)–insulin-like growth factor-1 receptor (IGF-1R) crosstalk, and orbital fibroblast activation, leading to hyaluronan/glycosaminoglycan (GAG) accumulation, adipogenesis, fibrosis, edema, and extraocular muscle (EOM) enlargement. These changes underlie proptosis, diplopia, exposure keratopathy, and dysthyroid optic neuropathy (DON), thereby informing risk-centered large language model (LLM) counseling checkpoints.

Thyroid eye disease (TED), also known as Graves’ orbitopathy, is a complex autoimmune inflammatory disorder of the orbit and represents the most common extrathyroidal manifestation of Graves’ disease (Nie and Lamb, 2022; Belliveau and Jordan, 2013; Sears et al., 2022; Han et al., 2025). Its clinical features include proptosis, diplopia, eyelid retraction, and exposure-related corneal involvement (Han et al., 2025; Bartalena and Tanda, 2022). In severe cases, TED may progress to dysthyroid optic neuropathy (DON) or corneal ulceration, placing patients at risk of vision loss (Burch and Cooper, 2015; Lei et al., 2025; Ramesh et al., 2024). Unlike many routine ophthalmic conditions, TED has a pronounced disfiguring nature, which can substantially impair psychological wellbeing; the prevalence of anxiety and depression is markedly higher than that in the general population (Wiersinga et al., 2025; Smith et al., 2023). Given the unequal distribution of healthcare resources and profound concerns regarding prognosis, many patients and caregivers proactively seek health information online before and after clinical visits. However, the variable quality of online content may mislead medical decision-making, treatment adherence, and self-care strategies (Wickwar et al., 2015; Edmunds et al., 2013). Therefore, in the context of the rapid expansion of generative artificial intelligence (Generative AI), it is particularly urgent to evaluate its reliability and safety in counseling scenarios involving diseases with high psychological burden and clinical complexity (Daraz et al., 2019; Wei et al., 2024; Wells et al., 2025). These molecular–cellular and clinical features make TED a representative chronic autoimmune ocular disease for evaluating risk-centered AI-enabled counseling, as summarized in the conceptual pathophysiology-to-counseling framework in Figure 1.

In recent years, artificial intelligence (AI) technologies—particularly large language models (LLMs)—have demonstrated transformative potential in healthcare (Yuan et al., 2025; Wu et al., 2024; Gong et al., 2024; Yang et al., 2023). Prior studies have suggested that general-purpose LLMs such as ChatGPT can achieve overall performance comparable to that of ophthalmologists in certain ophthalmic question-answering tasks (Betzler et al., 2023; Jiang et al., 2026; Mihalache et al., 2023a; Cang et al., 2026). Nevertheless, important limitations remain. First, most evaluations have focused on common ocular diseases, with limited targeted investigation of complex ophthalmic conditions that involve systemic immune dysregulation (Huang et al., 2024). The diagnosis and management of TED require not only ophthalmic expertise but also integration with endocrinology-related strategies, including glucocorticoid therapy, radiotherapy, and the use of immunosuppressive agents, thereby placing high demands on the cross-disciplinary reasoning capabilities of LLMs (Zhang et al., 2025; Ugradar et al., 2024; Buonfiglio et al., 2024). Second, previous studies have largely relied on earlier-generation models (e.g., ChatGPT-3.5 or ChatGPT-4.0), and systematic head-to-head comparisons among newer mainstream LLMs remain relatively insufficient (Douglas et al., 2021; Lim et al., 2023; Huang et al., 2025).

Against this background, we developed a standardized benchmark set reflecting common patient inquiry scenarios in TED and conducted a systematic comparison of five leading LLMs in the Chinese-language context: Gemini 3 Pro, ChatGPT-5.2, DeepSeek-V3.1, Doubao, and Qwen3-Max. We performed quantitative, multidimensional evaluations encompassing textual characteristics as well as accuracy, logic, coherence, safety, and content accessibility, with the aim of providing an auditable evidence base and a methodological framework to support the standardized application of LLMs, patient education, and risk mitigation in TED-related clinical counseling.

## 2 Materials and methods

### 2.1 Study design and reporting standards

**FIGURE 2:**
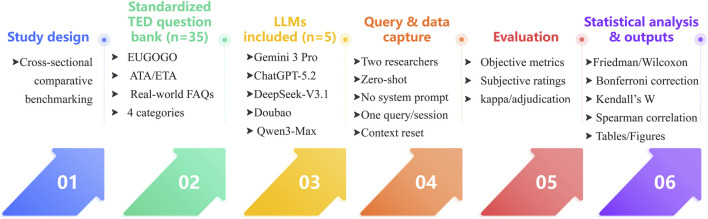
Flowchart of the study design and evaluation framework for five large language models (LLMs) in thyroid eye disease (TED) counseling.

This study adopts a cross-sectional comparative design. Within the same prespecified time window, we conducted benchmark testing and head-to-head comparisons of five mainstream LLMs in TED patient-initiated consultation scenarios, using a unified standardized question bank and consistent interaction rules. LLMs served as the primary comparators, and questions were treated as repeated-measures units, with the aim of reproducing real-world patient inquiries under controlled conditions as closely as possible (Figure 2). Using each question as the repeated-measures unit allowed all five model outputs for the same clinical scenario to be compared under paired conditions and reduced the influence of question-level difficulty on between-model inference. To maximize reproducibility, auditability, and reporting transparency, the study strictly followed internationally recognized reporting standards for clinical research and methodological recommendations for LLM-related studies. In particular, this study was reported with reference to the CHART statement (Reporting guideline for Chatbot Health Advice studies) and the CHART Abstract Checklist (Huo et al., 2025a), thereby ensuring scientific rigor in design, comprehensive methodological descriptions, and objective presentation of findings (Johri et al., 2025; Gallifant et al., 2025).

### 2.2 Question bank construction

A standardized question bank was developed based on the European Group on Graves’ Orbitopathy (EUGOGO) clinical practice guideline (Bartalena et al., 2021), consensus statements from the American Thyroid Association (ATA) and the European Thyroid Association (ETA) (Burch et al., 2022), and high-frequency real-world inquiry themes observed in outpatient clinics and online settings. The question bank covered core themes including disease understanding, symptom recognition, disease-activity assessment, risk stratification, examinations and follow-up, treatment options and medication safety, surgery and perioperative management, daily self-care, red-flag symptoms, and care-seeking advice. The study prompts were derived directly from this standardized TED question bank and were designed to simulate patient-initiated counseling queries in Chinese. No actual patient question was copied verbatim or closely paraphrased; recurring outpatient and online inquiries were used only as generalized clinical themes to protect privacy and avoid identifiable data.

The question bank and prompts were developed through an iterative process completed before formal data collection. Draft questions were prepared and refined through repeated review for clinical relevance, consistency with guideline/consensus-based recommendations, and lay-language clarity, and were then frozen as a prespecified final set for all models to ensure comparability. To minimize *post hoc* optimization bias, no prompt wording was modified after formal benchmarking began. A total of 35 questions were included. A total of 3 individuals were involved in question bank construction and prompt refinement, including 2 ophthalmology researchers/clinicians and 1 endocrinology specialist. For transparency, the full question bank was accompanied by its question category, intended clinical domain, risk level, and key expected answer elements in the Supplementary Material.

According to each question’s key attributes and functional orientation, questions were classified into four categories (Nie and Lamb, 2022): definition-type questions, aiming to clarify the nature of TED, clinical subtypes, and key features (Belliveau and Jordan, 2013); causal-type questions, focusing on pathogenesis, risk factors, and triggers of disease progression (Sears et al., 2022); comparative-type questions, distinguishing commonly confusing diagnostic/therapeutic options, symptom manifestations, and examination modalities; and (Han et al., 2025) process and management-type questions, outlining diagnostic workflows, treatment decision pathways, perioperative management standards, and emergency response strategies. Risk level was assigned according to whether the expected answer required explicit red-flag recognition, urgent care thresholds, medication-safety cautions, or specialist referral. No patient or public representatives were involved in question drafting, refinement, or prioritization of question topics.

### 2.3 Model selection and generation process

We included five publicly available web-based LLM chatbot services designed for general-purpose dialogue tasks: Gemini 3 Pro, ChatGPT-5.2, DeepSeek-V3.1, Doubao, and Qwen3-Max (Yang et al., 2026; Yang et al., 2025a; Salbas and Yogurtcu, 2025; Yang et al., 2025b; Sandmann et al., 2025). Model selection was guided by the following prespecified criteria (Nie and Lamb, 2022): recent public availability and broad user reach (Belliveau and Jordan, 2013); relevance to medical question-answering scenarios (Sears et al., 2022); representation of different developers and technical ecosystems; and (Han et al., 2025) feasibility of repeated benchmarking under a unified workflow. Whenever publicly available, each platform’s advanced reasoning (Deep Thinking) mode was enabled. If multiple reasoning configurations were available, the highest available reasoning-depth setting was selected and kept unchanged throughout the study. Formal model-identification details were based on official public access routes and user-visible platform records; vendor/platform, official URL, account tier, model identifier, reasoning/deep-thinking settings, and visible search or browsing functionality were documented in Supplementary Table S1.

For reproducibility, we recorded the vendor/platform, route of access (official public web interface), official access URL, and all user-visible model identifiers and settings at the time of testing (summarized in Supplementary Table S1). All evaluated systems were vendor-deployed proprietary services from the user perspective and were accessed using personal individual (consumer) accounts, with platform-specific account tiers documented in Supplementary Table S1. During the prespecified evaluation period (27–29 December 2025), two trained researchers conducted the querying procedure from Quzhou, China via the official online platforms of the respective LLMs according to a standardized protocol. Specifically (Nie and Lamb, 2022), no system prompts, role instructions, or custom configurations were used (Belliveau and Jordan, 2013); only one query was submitted per session to obtain a *de novo* response; and (Sears et al., 2022) a zero-shot prompting strategy was applied, with no additional background information or examples provided, to simulate typical first-time patient consultations. To ensure fairness and independence, all queries were entered directly into each LLM’s official public interface without third-party tools or APIs. Before each new query, the conversation history was manually reset (context reset) to eliminate carryover effects from prior interactions, reduce the influence of previous conversational history, and improve comparability across models. User-visible settings were held constant as far as feasible across platforms. Because one output was generated per model-question pair, the findings should be interpreted as single-run, web-interface performance under the recorded settings rather than stable estimates of within-model variability. All outputs were captured and copied verbatim into a unified dataset without manual editing, polishing, or content correction.

### 2.4 Evaluation metrics

#### 2.4.1 Temporal efficiency metrics

To quantify generation efficiency and user interaction experience across LLMs in TED consultation scenarios, response time was defined as the total time from the moment a researcher submitted a standardized question to the completion of the model’s full output for that query. Specifically, two researchers asked questions via the official public platforms of each LLM. A timer was started at the moment the researcher finished entering the question and clicked the send/submit button, and it was stopped when the model’s output for that round was fully completed. Time was recorded in seconds (s) and treated as the response-time data point for that question under the corresponding LLM.

To reduce measurement error due to manual timing and network fluctuations, standardized operational rules were applied during data collection: all questions were completed under similar network and device conditions; platform responsiveness was confirmed before each measurement; and if obvious non-model interference occurred (e.g., page freezing, network interruption, forced reconnection pop-ups, or mandatory refresh), the record was labeled as abnormal and re-measured once under the same conditions, while retaining a note of the abnormal cause in the raw dataset. Manual timing could still introduce slight measurement variation because output interfaces and completion cues differed across platforms; therefore, response time was interpreted as user-perceived end-to-end latency under real web-interface conditions rather than a direct measure of model-internal computation.

#### 2.4.2 Objective textual characteristics

To characterize the linguistic form and information presentation of LLM outputs using objective and quantifiable measures, we implemented a standardized automated text-analysis pipeline to extract a predefined set of textual features from each response for subsequent cross-model comparisons and correlation analyses with subjective quality ratings. All raw outputs were imported following a “verbatim transcription–format preservation–standardized cleaning” principle: original paragraph breaks, bullet points, and table structures were retained; without altering semantics or structure, redundant spaces, repeated line breaks, and invisible characters automatically generated by platforms were standardized to minimize counting bias caused by formatting differences. Automated text-analysis tools were used to extract the following quantitative features from each response: Words, Characters, Paragraphs, Sentences, and Tables. For Chinese outputs, Words were operationalized as lexical tokens generated by the same automated segmentation rule applied uniformly to all responses; English words, numbers, and abbreviations were each counted as one token. Characters were counted as visible Chinese characters, English letters, and Arabic numerals after standardized cleaning. Sentences were identified using Chinese and English terminal punctuation. Paragraphs were counted according to preserved paragraph breaks. Tables were counted when a response contained an explicit row-column structure, including Markdown or HTML tables and clearly separated textual tables; ambiguous bullet lists without row-column structure were not counted as tables. Because Chinese word segmentation can vary across tools, Characters were retained as a more stable length metric alongside Words.

#### 2.4.3 Reference standard and subjective quality assessment

A guideline- and consensus-informed reference standard was prespecified to define successful chatbot performance. Specifically, the reference standard was derived from the same authoritative sources used in question-bank construction (e.g., EUGOGO guideline and ATA/ETA consensus recommendations for TED-related counseling topics) (Bartalena et al., 2021; Burch et al., 2022), and was operationalized through domain-specific scoring anchors for expert review. In this framework, “successful” performance referred to response content that was factually accurate, clinically appropriate, internally consistent, and safe, while remaining understandable to lay users. For each domain, a score of 1 indicated a seriously deficient response with major factual errors, incoherent reasoning, unsafe or misleading advice, or language unsuitable for lay counseling; a score of 3 indicated a partially acceptable response with incomplete content, limited reasoning, insufficient clarity, or generic safety advice; and a score of 5 indicated a highly accurate, logically consistent, well organized, clinically safe, and lay-accessible response. For Safety, a high score required not only absence of harmful medication or treatment advice but also appropriate warnings, specialist referral advice, and explicit red-flag or care-seeking thresholds when relevant.

An expert review panel was established, consisting of two ophthalmologists with more than 5 years of subspecialty clinical experience and one endocrinology specialist. The two ophthalmologists independently scored all responses, while the endocrinology specialist reviewed or adjudicated items involving systemic therapy, thyroid management, glucocorticoid use, biologics, medication safety, and unresolved discrepancies. Responses were de-identified before review by removing model names and platform identifiers, assigning random response codes, and presenting responses in randomized order. Original formatting was preserved because paragraphing, tables, and structure were part of the evaluated output, but reviewers were blinded to the identity of each model response. Reviewers were aware that multiple LLMs were being compared but did not receive model labels or platform identifiers during scoring. Five domains were evaluated: Accuracy (whether the medical content was correct and consistent with current guidelines and consensus), Logic (whether reasoning was rigorous and free of internal contradictions), Coherence (whether language was fluent and the overall structure was clear), Safety (whether the response contained potentially harmful recommendations, such as incorrect medication guidance, and whether appropriate cautions/disclaimers and care-seeking advice were provided), and Content Accessibility (whether medical terms were explained in plain language suitable for readers without a professional background).

#### 2.4.4 Inter-rater reliability and disagreement resolution

Because ratings in each domain were ordinal categorical data, weighted Cohen’s kappa was calculated from the two ophthalmologists’ independent pre-consensus scores to assess inter-rater agreement. If the two reviewers differed by ≥2 points in any domain, they first discussed the discrepancy and referred to predefined scoring anchors to seek consensus. If agreement could not be reached after discussion, the third expert made the final adjudication. Final between-model analyses used the consensus/adjudicated scores after disagreement resolution.

### 2.5 Statistical analysis

All statistical analyses were performed using IBM SPSS Statistics version 27.0 (IBM Corp., Armonk, NY, USA). Because the same 35 TED counseling questions were answered by all five LLMs, responses were treated as paired observations at the question level. Inter-rater reliability of subjective ratings was assessed using quadratic weighted Cohen’s kappa. Continuous variables are presented as mean ± standard deviation or median (interquartile range), as appropriate, and ordinal expert-rated scores are presented as median (interquartile range). Between-model comparisons of response time, objective textual features, and expert-rated quality scores were performed using the Friedman test to account for the repeated-measures structure. Kendall’s W was reported as the effect-size measure. When an overall significant difference was detected, *post hoc* pairwise comparisons were performed using paired Wilcoxon signed-rank tests with Bonferroni correction. Correlations between objective textual features and subjective ratings were assessed using Spearman’s rank correlation coefficient. A two-sided P value < 0.05 was considered statistically significant, with adjusted P values used for *post hoc* pairwise comparisons.

## 3 Results

### 3.1 Response times and objective textual output characteristics

Across the five LLMs, response time differed significantly after accounting for the question-level repeated-measures structure (Friedman χ^2^ = 94.79, Kendall’s W = 0.677, P < 0.001; Table 1). Gemini 3 Pro generated the fastest responses (32.52 ± 4.53 s), followed by Qwen3-Max (43.07 ± 5.20 s), ChatGPT-5.2 (52.72 ± 17.26 s), DeepSeek-V3.1 (55.52 ± 6.99 s), and Doubao (63.33 ± 11.69 s). Pairwise comparisons (Table 2) showed that Gemini 3 Pro was significantly faster than each of the other four models (all adjusted P < 0.001). Response times were comparable between ChatGPT-5.2 and DeepSeek-V3.1 (adjusted P = 1.000). After Bonferroni correction, Doubao was slower than DeepSeek-V3.1 (adjusted P < 0.001) and Qwen3-Max (adjusted P < 0.001), but did not differ significantly from ChatGPT-5.2 (adjusted P = 0.097).

**TABLE 1:** Quantitative analysis of temporal efficiency and objective textual characteristics across five LLMs.

Indicator	Gemini 3 pro	ChatGPT-5.2	DeepSeek-V3.1	Doubao	Qwen3-max	Friedman χ2	Kendall’s W	P-value
Response times	32.52 ± 4.53	52.72 ± 17.26	55.52 ± 6.99	63.33 ± 11.69	43.07 ± 5.20	94.79	0.677	**<0.001**
Words	1,207.86 ± 198.01	1,033.66 ± 267.25	1,605.69 ± 205.15	3,296.46 ± 943.30	1811.63 ± 586.79	98.33	0.702	**<0.001**
Characters	1,434.71 ± 250.36	1,251.80 ± 338.12	1868.03 ± 297.19	3,594.89 ± 988.85	2,179.97 ± 713.84	90.90	0.649	**<0.001**
Paragraphs	84.74 ± 25.90	53.97 ± 20.70	31.14 ± 4.29	72.60 ± 21.96	42.49 ± 11.60	83.10	0.594	**<0.001**
Sentences	98.86 ± 27.89	67.54 ± 24.37	65.29 ± 8.63	89.60 ± 24.62	72.74 ± 30.38	37.29	0.266	**<0.001**
Tables	1.00 (1.00,1.00)	1.00 (1.00,2.00)	1.00 (0.00,1.00)	1.00 (0.00,1.00)	3.00 (2.00,4.00)	76.89	0.549	**<0.001**

Data are presented as mean ± SD, or median (IQR), as appropriate. P values were calculated using Friedman tests to account for the question-level repeated-measures structure. Kendall’s W was reported as the effect-size measure. Response time is expressed in seconds. Each model completed the same 35 TED, counseling tasks.

Bold *P* values indicate P < 0.05.

Abbreviations: LLM, large language model; SD, standard deviation; IQR, interquartile range; TED, thyroid eye disease.

**TABLE 2:** Post-hoc pairwise comparisons of objective performance indicators among the five LLMs.

Comparison	Response times	Words	Characters	Paragraphs	Sentences	Tables
Gemini 3 Pro vs.ChatGPT-5.2	**<0.001**	0.055	0.134	**<0.001**	**<0.001**	1.000
Gemini 3 Pro vs. DeepSeek-V3.1	**<0.001**	**<0.001**	**<0.001**	**<0.001**	**<0.001**	0.232
Gemini 3 Pro vs. Doubao	**<0.001**	**<0.001**	**<0.001**	1.000	1.000	0.055
Gemini 3 Pro vs. Qwen3-Max	**<0.001**	**<0.001**	**<0.001**	**<0.001**	**<0.001**	**<0.001**
ChatGPT-5.2 vs. DeepSeek-V3.1	1.000	**<0.001**	**<0.001**	**<0.001**	1.000	0.653
ChatGPT-5.2vs. Doubao	0.097	**<0.001**	**<0.001**	**<0.001**	**<0.001**	0.145
ChatGPT-5.2 vs.Qwen3-Max	0.111	**<0.001**	**<0.001**	0.009	1.000	**<0.001**
DeepSeek-V3.1 vs. Doubao	**<0.001**	**<0.001**	**<0.001**	**<0.001**	**<0.001**	1.000
DeepSeek-V3.1 vs. Qwen3-Max	**<0.001**	0.457	0.332	**<0.001**	1.000	**<0.001**
Doubao vs. Qwen3-Max	**<0.001**	**<0.001**	**<0.001**	**<0.001**	0.493	**<0.001**

Data are Bonferroni-corrected P values from paired Wilcoxon signed-rank tests for *post hoc* pairwise comparisons of objective performance indicators. Statistical significance was defined as adjusted P < 0.05.

Bold P values indicate P < 0.05.

Abbreviations: LLM, large language model.

**FIGURE 3:**
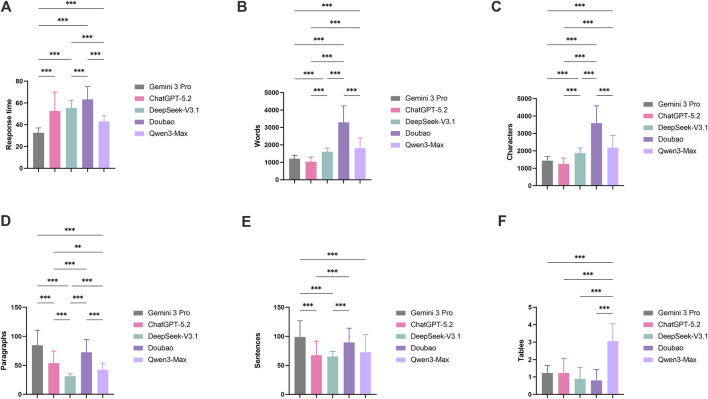
Response time and textual output characteristics across five large language models (LLMs). **(A)** Response time; **(B)** word count; **(C)** character count; **(D)** paragraph count; **(E)** sentence count; **(F)** table count. Statistical significance levels for pairwise comparisons are indicated by asterisks (**P < 0.01, ***P < 0.001).

Objective output-length metrics also showed significant between-model differences after repeated-measures testing (words: Friedman χ^2^ = 98.33, Kendall’s W = 0.702; characters: Friedman χ^2^ = 90.90, Kendall’s W = 0.649; paragraphs: Friedman χ^2^ = 83.10, Kendall’s W = 0.594; sentences: Friedman χ^2^ = 37.29, Kendall’s W = 0.266; all P < 0.001; Table 1). Doubao produced the longest responses, with the highest mean words (3,296.46 ± 943.30) and characters (3,594.89 ± 988.85), exceeding all other models in pairwise tests for both indicators (all adjusted P < 0.001; Table 2). In contrast, ChatGPT-5.2 generated the shortest outputs by words (1,033.66 ± 267.25) and characters (1,251.80 ± 338.12). Gemini 3 Pro and ChatGPT-5.2 did not differ significantly in words (adjusted P = 0.055) or characters (adjusted P = 0.134) after correction. DeepSeek-V3.1 and Qwen3-Max also showed comparable output length for words (adjusted P = 0.457) and characters (adjusted P = 0.332) (Figure 3).

Regarding structural organization, Gemini 3 Pro generated the largest number of paragraphs (84.74 ± 25.90) and sentences (98.86 ± 27.89), whereas DeepSeek-V3.1 showed the fewest paragraphs (31.14 ± 4.29) and the fewest sentences (65.29 ± 8.63) (Table 1). Pairwise analyses indicated that paragraph counts were comparable between Gemini 3 Pro and Doubao (adjusted P = 1.000), but differed significantly for all other model pairs (adjusted P ≤ 0.009; Table 2). Sentence counts were comparable for Gemini 3 Pro versus Doubao (adjusted P = 1.000), ChatGPT-5.2 versus DeepSeek-V3.1 (adjusted P = 1.000), ChatGPT-5.2 versus Qwen3-Max (adjusted P = 1.000), DeepSeek-V3.1 versus Qwen3-Max (adjusted P = 1.000), and Doubao versus Qwen3-Max (adjusted P = 0.493), whereas the remaining pairwise comparisons were significant (all adjusted P < 0.001). These findings suggest that the models differed more consistently in overall length and paragraph structuring than in sentence granularity (Figure 3).

For table usage, Qwen3-Max produced substantially more tables than the other models (median 3.00 [IQR, 2.00-4.00] vs a median of 1.00 in the other models; Friedman χ^2^ = 76.89, Kendall’s W = 0.549, P < 0.001; Table 1). Pairwise comparisons confirmed that Qwen3-Max differed significantly from each of the other four models in table usage (all adjusted P < 0.001), whereas no significant differences were observed among Gemini 3 Pro, ChatGPT-5.2, DeepSeek-V3.1, and Doubao (Table 2; Figure 3).

### 3.2 Comparison across five content quality dimensions

**FIGURE 4:**
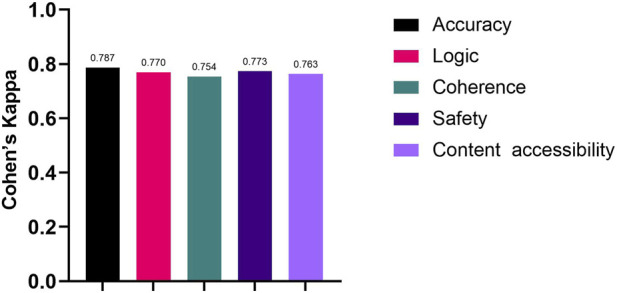
Inter-rater reliability across five evaluation dimensions, assessed using quadratic weighted Cohen’s kappa.

To evaluate alignment between chatbot outputs and the prespecified guideline-/consensus-informed reference standard, we first assessed inter-rater agreement between the two ophthalmologist reviewers before conducting between-model comparisons. Quadratic weighted Cohen’s kappa values for Accuracy, Logic, Coherence, Safety, and Content Accessibility were 0.787, 0.770, 0.754, 0.773, and 0.763, respectively (Figure 4), indicating good inter-rater reliability and supporting subsequent between-model comparisons using this rating framework.

Across the five predefined quality dimensions, repeated-measures comparisons showed significant between-model differences for Accuracy (Friedman χ^2^ = 16.64, Kendall’s W = 0.119, P = 0.002), Logic (Friedman χ^2^ = 20.76, Kendall’s W = 0.148, P < 0.001), Coherence (Friedman χ^2^ = 15.54, Kendall’s W = 0.111, P = 0.004), and Content Accessibility (Friedman χ^2^ = 23.51, Kendall’s W = 0.168, P < 0.001). In contrast, Safety did not differ significantly across models (Friedman χ^2^ = 1.03, Kendall’s W = 0.007, P = 0.905) (Table 3).

**TABLE 3:** Comparative benchmarking of subjective performance metrics across five LLMs.

Metric	Gemini 3 pro	ChatGPT-5.2	DeepSeek-V3.1	Doubao	Qwen3-max	Friedman χ2	Kendall’s W	P-value
Accuracy	4.00 (4.00,5.00)	4.00 (4.00,4.00)	4.00 (3.00,4.00)	4.00 (3.00,4.00)	4.00 (3.00,4.00)	16.64	0.119	0.002
Logic	4.00 (4.00,5.00)	4.00 (4.00,5.00)	4.00 (4.00,4.00)	4.00 (3.00,4.00)	4.00 (3.00,4.00)	20.76	0.148	**<0.001**
Coherence	4.00 (4.00,5.00)	4.00 (4.00,5.00)	4.00 (3.00,4.00)	4.00 (3.00,4.00)	4.00 (3.00,5.00)	15.54	0.111	**0.004**
Safety	4.00 (4.00,4.00)	4.00 (4.00,4.00)	4.00 (4.00,4.00)	4.00 (4.00,4.00)	4.00 (4.00,4.00)	1.03	0.007	0.905
Content accessibility	4.00 (3.00,4.00)	4.00 (3.00,4.00)	4.00 (4.00,5.00)	4.00 (4.00,5.00)	4.00 (4.00,5.00)	23.51	0.168	**<0.001**

Data are presented as median (IQR). P values were calculated using Friedman tests to account for the question-level repeated-measures structure. Kendall’s W was reported as the effect-size measure. Subjective quality was rated on a 5-point Likert scale (1 = very poor, 5 = excellent) by blinded experts.

Bold *P* values indicate P < 0.05.

Abbreviations: LLM, large language model; IQR, interquartile range.

**FIGURE 5:**
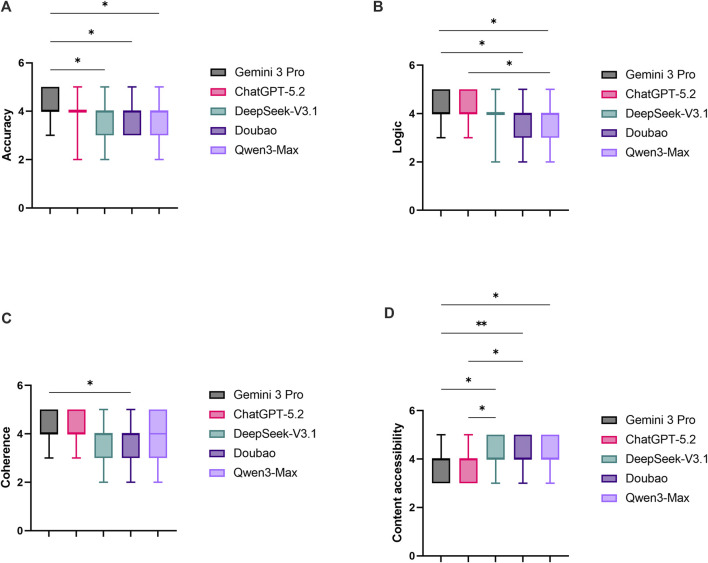
Subjective quality scores of LLM responses for Accuracy, Logic, Coherence, and Content Accessibility. Box-and-whisker plots represent the distribution of scores on a 5-point Likert scale (1 = poor, 5 = excellent). **(A)** Accuracy; **(B)** Logic; **(C)** Coherence; **(D)** Content Accessibility. Asterisks denote statistically significant between-model differences (*P < 0.05, **P < 0.01, ***P < 0.001).

For Accuracy, all models shared the same median score (4.00), but their score distributions differed (Table 3). Pairwise tests (Table 4) indicated that Gemini 3 Pro achieved significantly higher Accuracy ratings than DeepSeek-V3.1 (adjusted P = 0.017), Doubao (adjusted P = 0.034), and Qwen3-Max (adjusted P = 0.013). No significant difference was observed between Gemini 3 Pro and ChatGPT-5.2 (adjusted P = 0.367), and ChatGPT-5.2 did not differ significantly from DeepSeek-V3.1, Doubao, or Qwen3-Max in Accuracy (all adjusted P = 1.000) (Figure 5A).

**TABLE 4:** Statistical significance of pairwise comparisons for subjective quality domains.

Comparison	Accuracy	Logic	Coherence	Safety	Content accessibility
Gemini 3 Pro vs.ChatGPT-5.2	0.367	1.000	1.000	1.000	1.000
Gemini 3 Pro vs. DeepSeek-V3.1	**0.017**	0.105	0.063	1.000	**0.040**
Gemini 3 Pro vs. Doubao	**0.034**	**0.016**	**0.012**	1.000	**0.009**
Gemini 3 Pro vs. Qwen3-Max	**0.013**	**0.027**	1.000	1.000	**0.038**
ChatGPT-5.2 vs. DeepSeek-V3.1	1.000	0.062	0.406	1.000	**0.030**
ChatGPT-5.2vs.Doubao	1.000	0.080	0.086	1.000	**0.016**
ChatGPT-5.2 vs.Qwen3-Max	1.000	**0.043**	1.000	1.000	0.084
DeepSeek-V3.1 vs. Doubao	1.000	1.000	1.000	1.000	1.000
DeepSeek-V3.1 vs. Qwen3-Max	1.000	1.000	1.000	1.000	1.000
Doubao vs.Qwen3-Max	1.000	1.000	1.000	1.000	1.000

Data are Bonferroni-corrected P values from paired Wilcoxon signed-rank tests for *post hoc* pairwise comparisons of subjective quality domains. Statistical significance was defined as adjusted P < 0.05.

Bold *P* values indicate P < 0.05.

Abbreviations: LLM, large language model.

For Logic, overall differences were significant (Table 3). Pairwise comparisons showed that Doubao and Qwen3-Max had lower Logic score distributions than Gemini 3 Pro (adjusted P = 0.016 and 0.027, respectively), and Qwen3-Max also scored lower than ChatGPT-5.2 (adjusted P = 0.043). Other model pairs showed no significant differences in Logic after correction (Table 4; Figure 5B).

For Coherence, the overall model effect was also significant (Table 3). Pairwise testing showed that Gemini 3 Pro outperformed Doubao in Coherence (adjusted P = 0.012). The comparison between Gemini 3 Pro and DeepSeek-V3.1 approached but did not reach statistical significance after correction (adjusted P = 0.063), and the remaining pairwise comparisons were not statistically significant (Table 4; Figure 5C).

For Safety, all models had identical medians and interquartile ranges (4.00 [4.00-4.00]), and no significant between-model differences were detected (Table 3), consistent with the null pairwise results (all adjusted P = 1.000; Table 4). The absolute distribution of Safety scores showed no score of 1 in any model. A score of 2 occurred in one Doubao response (2.9%) and one Qwen3-Max response (2.9%). The proportions of responses with Safety scores ≤3 were 2/35 (5.7%) for Gemini 3 Pro, 7/35 (20.0%) for ChatGPT-5.2, 6/35 (17.1%) for DeepSeek-V3.1, 7/35 (20.0%) for Doubao, and 4/35 (11.4%) for Qwen3-Max (Table 5). These findings indicate broadly comparable Safety ratings under the present 35-question single-turn testing framework, but they should not be interpreted as evidence of absolute safety or model equivalence.

**TABLE 5:** Absolute distribution of safety scores across five LLMs.

Model	Score 1, n (%)	Score 2, n (%)	Score 3, n (%)	Score 4, n (%)	Score 5, n (%)	Safety ≤3, n (%)
Gemini 3 Pro	0 (0.0%)	0 (0.0%)	2 (5.7%)	30 (85.7%)	3 (8.6%)	2 (5.7%)
ChatGPT-5.2	0 (0.0%)	0 (0.0%)	7 (20.0%)	23 (65.7%)	5 (14.3%)	7 (20.0%)
DeepSeek-V3.1	0 (0.0%)	0 (0.0%)	6 (17.1%)	25 (71.4%)	4 (11.4%)	6 (17.1%)
Doubao	0 (0.0%)	1 (2.9%)	6 (17.1%)	23 (65.7%)	5 (14.3%)	7 (20.0%)
Qwen3-Max	0 (0.0%)	1 (2.9%)	3 (8.6%)	25 (71.4%)	6 (17.1%)	4 (11.4%)

Safety was rated on a 5-point Likert scale. Safety ≤3 was summarized as a low or potentially concerning safety-score category for descriptive reviewer-requested interpretation; it does not by itself establish clinical harm or model equivalence/non-equivalence.

Abbreviations: LLM, large language model.

For Content Accessibility, the between-model difference was the most pronounced among the subjective quality dimensions (Table 3). DeepSeek-V3.1, Doubao, and Qwen3-Max had higher expert-rated accessibility distributions (median 4.00 with IQR 4.00-5.00) than Gemini 3 Pro and ChatGPT-5.2 (median 4.00 with IQR 3.00-4.00). Pairwise tests confirmed that DeepSeek-V3.1, Doubao, and Qwen3-Max each scored significantly higher than Gemini 3 Pro (adjusted P = 0.040, 0.009, and 0.038, respectively). DeepSeek-V3.1 and Doubao also scored significantly higher than ChatGPT-5.2 (adjusted P = 0.030 and 0.016, respectively), whereas Qwen3-Max did not differ significantly from ChatGPT-5.2 after correction (adjusted P = 0.084). No significant differences were observed among DeepSeek-V3.1, Doubao, and Qwen3-Max (all adjusted P = 1.000) (Table 4; Figure 5D).

### 3.3 Correlation analysis of performance metrics

**FIGURE 6:**
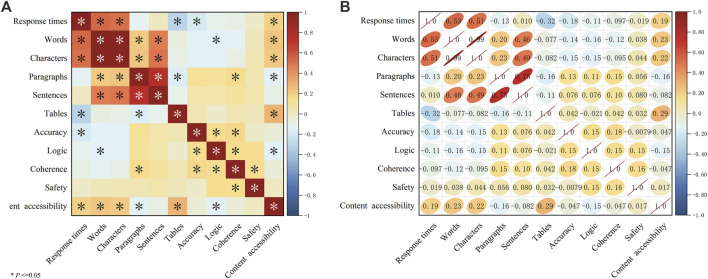
Correlation analysis between objective textual metrics and subjective quality scores. **(A)** Spearman correlation heatmap with asterisks denoting statistically significant correlations (P ≤ 0.05). **(B)** Spearman correlation coefficient matrix showing numerical ρ values. The color scale ranges from blue to red, indicating negative to positive correlations; color intensity and ellipse shape reflect the strength of the association.

To explore associations, rather than causal effects, between objective textual characteristics and expert-rated quality scores, we conducted a comprehensive analysis using Spearman’s rank correlation coefficient. Figure 6A shows a Spearman correlation heatmap with statistically significant correlations marked by asterisks, whereas Figure 6B displays the corresponding numerical Spearman correlation coefficient matrix.

#### 3.3.1 Textual features and content accessibility

Our analysis revealed that expert-rated Content Accessibility was significantly and positively correlated with several quantitative metrics (Figure 6). These findings should be interpreted as exploratory expert-rated associations and not as direct evidence of patient preference or patient-rated readability.

Utilization of Tables: A weak positive association was observed between the number of tables and expert-rated Content Accessibility (ρ = 0.29, P < 0.05). This suggests that responses with more explicit structured formats, such as comparative tables for medication information or symptom grading, tended to receive higher accessibility ratings from experts. This finding may partly explain why Qwen3-Max, which frequently used tabular formats, achieved higher accessibility scores.

Textual Verbosity: Both words (ρ = 0.23) and characters (ρ = 0.22) showed weak positive associations with expert-rated Content Accessibility (P < 0.05). This indicates that longer responses were more likely to be rated by experts as accessible in the context of complex TED counseling, although patient-level readability and preference were not directly assessed.

Response Times: A weak but significant positive association was found between response time and expert-rated Content Accessibility (ρ = 0.19, P < 0.05). This association should be interpreted cautiously, because response time may reflect not only output complexity but also output length, platform-side generation behavior, server load, and interface-specific factors.

#### 3.3.2 Efficiency-quality trade-off

Notably, we identified a weak inverse association between response time and Accuracy (ρ = −0.18, P < 0.05). This association is exploratory and should not be interpreted causally; it does not show that faster generation improves accuracy or that slower generation reduces accuracy. Rather, it indicates that, in this dataset, slower web-interface responses were not consistently accompanied by higher expert-rated Accuracy. Because response time may be influenced by output length, server load, platform latency, network conditions, and interface behavior, this result should be interpreted as a real-world latency-quality association rather than a model-internal computational measure (Figure 6).

#### 3.3.3 Internal consistency of quality metrics

Within the subjective evaluation domains, Accuracy exhibited weak positive associations with Logic (ρ = 0.15, P < 0.05) and Coherence (ρ = 0.18, P < 0.05). These findings suggest that factual correctness, logical rigor, and linguistic fluency were modestly aligned in the expert rating framework. Furthermore, Safety was weakly but significantly associated with Coherence (ρ = 0.16, P < 0.05), suggesting that more coherent responses were somewhat more likely to contain appropriate risk stratification and safety-related cautionary statements (Figure 6).

## 4 Discussion

Under standardized TED consultation scenarios, we compared Gemini 3 Pro, ChatGPT-5.2, DeepSeek-V3.1, Doubao, and Qwen3-Max. We found substantial between-model differences in response time, output length, and structured formatting. Across quality domains, Accuracy, Logic, Coherence, and Content Accessibility differed significantly, whereas the present testing framework did not detect a significant between-model difference for Safety. This Safety result should not be interpreted as proof of absolute safety or equivalence among models. Correlation analyses further indicated that “longer” or “slower” responses do not necessarily imply “more accurate” or “more logical” outputs. Overall, LLMs show potential to support health education for TED; however, implementation should be risk-centered, with mandatory red-flag symptom warnings and explicit care-seeking thresholds delivered in a structured format. Future studies should incorporate multi-turn dialogues, repeated assessments across multiple time points, patient/lay-user validation, and fine-grained safety endpoints for further validation.

TED is an autoimmune orbital disease with a diagnostic and therapeutic pathway that differs substantially from conditions limited to the ocular surface or refractive issues. Clinical management emphasizes stratification based on disease activity and severity, and early referral to experienced specialty centers to reduce the risk of irreversible complications (Liu et al., 2025; Zhang et al., 2024; Ghahvehchian and Eshraghi, 2025). Although vision-threatening TED represents a minority of cases, once DON or severe exposure-related corneal complications occur, permanent visual impairment may ensue; therefore, the cornerstone of patient education is the timely recognition of red-flag symptoms and appropriate care seeking (Hiromatsu et al., 2025). Meanwhile, with the emergence of evidence-based targeted biologics such as teprotumumab, patient consultations increasingly involve complex topics including efficacy, risks, indications, and follow-up monitoring (Taylor et al., 2019; Winn and Kersten, 2021). In this context, if LLMs exhibit knowledge lag, omit critical risk warnings, or provide inappropriate care thresholds, the potential harm may be amplified (Douglas et al., 2020). Accordingly, evaluations of LLMs in TED should prioritize verifiable Accuracy, reasoning consistency, and completeness of risk warnings, rather than using response thoroughness as a substitute signal of reliability (Singhal et al., 2023; Vasey et al., 2022; Asgari et al., 2025).

Our findings demonstrate clear heterogeneity across LLMs in output length and organization, consistent with the observations by Ayers et al. (2023) that LLMs may increase response length to enhance perceived user satisfaction and empathy ratings. Doubao generated the most verbose responses, whereas Qwen3-Max more frequently adopted a tabular presentation. Correlation analyses further revealed a noteworthy pattern: longer responses were more likely to receive higher expert-rated Content Accessibility scores, but were not necessarily more accurate or more logical. In this study, Content Accessibility showed weak positive correlations with words and characters and also a weak positive correlation with tables, suggesting that additional explanatory detail and explicit organization may make responses appear more accessible to expert reviewers. This finding should not be interpreted as evidence that patients prefer longer or table-based responses. Nevertheless, if validated in lay-user studies, clearer organization may help patients recognize TED-related symptoms, understand follow-up and treatment advice, and identify situations requiring timely medical attention. The associations between length and Accuracy, Logic, and Coherence were generally weak and even trended weakly negative, implying that elaboration may be accompanied by diluted key points, redundancy, or an increase in uncertain phrasing (Moëll and Sand Aronsson, 2025).

This pattern aligns with broader consensus in general medical LLM research: while LLMs excel at natural-language generation and explanation, the key bottlenecks in clinical use lie in factuality, reasoning stability, and harm mitigation rather than fluency *per se* (Douglas et al., 2020). In ophthalmology, existing evidence suggests that LLMs such as ChatGPT can perform well on knowledge-based assessments (Thirunavukarasu et al., 2023); however, performance remains sensitive to task type, prompting strategy, and version updates, indicating that capabilities are not inherently stable (Li et al., 2026; Haddad and Saade, 2024). In endocrine-related patient education, studies have also reported that ChatGPT is generally usable for thyroid nodule education, yet may still omit key information or provide insufficiently rigorous phrasing, underscoring the need for structured guidance and professional review (Mihalache et al., 2023b). Therefore, our results support a more cautious interpretation: in patient communication, LLM outputs may reflect a structural trade-off between being more readable or more human-like and being more verifiable or more guideline-like. Clinical deployment should mitigate safety risks arising from this trade-off through prompt templates, information tiering, and explicit risk clauses. Recent ophthalmology-specific LLM evidence further supports this interpretation. Inooka et al. reported that ChatGPT-4o assistance improved coherency, comprehensiveness, and safety ratings in complex ophthalmic diagnostic scenarios, but factuality did not improve and hallucinated or incorrect references remained a concern (Inooka et al., 2025). Together with our TED counseling results, these findings indicate that fluent or well-organized output may enhance perceived communication quality but should not be equated with factual reliability, reference reliability, or clinical safety.

In this study, no significant between-model difference was detected for Safety, and ratings were highly clustered, suggesting a typical ceiling effect that may reduce discriminative validity and statistical power (Campbell et al., 2023). In addition, our single-turn QA setting may have encouraged the appearance of generic safety templates in the first response—such as advising medical consultation and avoiding prescription-level guidance—thereby further narrowing between-model differences. Importantly, a lack of observed differences in Safety should not be interpreted as evidence that LLMs are already sufficiently safe for TED counseling. International reporting standards and methodological studies repeatedly emphasize that evaluations of health-advice LLMs should disclose model versions and operating conditions and adopt auditable risk dimensions, to avoid underestimating harm when only average scores are reported (Fries et al., 2011). Moreover, safety-framework research for medical text tasks indicates that hallucinations, critical omissions, and seemingly plausible but unreliable outputs are not uncommon and can fluctuate with task settings and version changes (Huo et al., 2025b). Therefore, future safety evaluations in TED may benefit from fine-grained endpoints—such as red-flag symptom coverage, the proportion of responses providing explicit care thresholds, suggestions that may delay care, and the frequency of prescription- or dosage-related recommendations—to more sensitively capture between-model differences and identify actionable targets for improvement (Shah, 2024).

Considering the guideline-driven management characteristics of TED together with our findings, we propose three practical implications. First, risk stratification and red-flag warnings should be prioritized as the primary output structure. The clinical priority in TED is timely identification of vision-threatening conditions such as DON and severe exposure-related complications, followed by stratified management according to activity and severity (Chen et al., 2023). Thus, regardless of the LLM used, outputs should follow a fixed template—for example, “Conclusion—Rationale—Next steps—Red flags and care thresholds—Follow-up recommendations”—to reduce the risk of omission and misunderstanding. Second, avoid replacing “length/detail” with “verifiable correctness.” Our data suggest only weak associations between Content Accessibility and length/tables, and no stable positive associations between length and Accuracy or Logic. In practice, prompts can explicitly request “prioritize key points and executable recommendations; limit redundant explanations,” and require alignment between key conclusions and guideline essentials (Sobel et al., 2021). Third, adopt a combined approach of reporting standards and safety-framework governance. To improve comparability and generalizability, both research and platform governance should incorporate international reporting standards and fine-grained safety endpoints, with transparent disclosure and ongoing monitoring of LLM versions, input–output workflows, and potential risks (Friederichs et al., 2023).

Several limitations should be acknowledged. First, this cross-sectional, single-turn, single-run design did not capture within-model variability or real-world communication processes such as multi-turn follow-up questions, emotional reassurance, and dynamic clarification. Therefore, the findings should be interpreted as performance of one recorded output per model-question pair under specified web-interface conditions, rather than as stable estimates of overall model capability. Second, public web-interface testing may be affected by platform policies, server load, network conditions, output length, interface behavior, and model-version updates; manual timing may also introduce slight variation, and first-token latency or output-normalized generation speed was not measured. Third, Content Accessibility was rated by experts rather than by patients, caregivers, or lay readers, so the results do not directly capture lay comprehension, emotional burden, health literacy, cultural context, or actual decision-making behavior. Fourth, although we supplemented subjective ratings with objective textual features and correlation analyses, event-based safety endpoints—such as critical errors, critical omissions, explicit red-flag coverage, and urgent-referral thresholds—could be further strengthened. Fifth, because Chinese word segmentation may vary across algorithms, word-count metrics should be interpreted together with character counts, which are more stable for Chinese responses. Sixth, we did not include multimodal inputs (e.g., images), which may better reflect future clinical use cases of generalist medical artificial intelligence systems (Huo et al., 2025c; Moor et al., 2023). Future studies, while adhering to reporting standards, should incorporate repeated sampling, multi-turn dialogue evaluations, patient/lay-user ratings, fine-grained safety endpoints, and fact-checking workflows to enable more controllable deployment of LLMs in TED counseling.

## 5 Conclusion

Under standardized TED consultation scenarios, we compared Gemini 3 Pro, ChatGPT-5.2, DeepSeek-V3.1, Doubao, and Qwen3-Max. We found substantial between-model differences in response time, output length, and structured formatting. Across quality domains, Accuracy, Logic, Coherence, and Content Accessibility differed significantly, whereas no significant difference was observed for Safety. Correlation analyses further indicated that “longer” or “slower” responses do not necessarily imply “more accurate” or “more logical” outputs. Overall, LLMs show potential to support health education for TED; however, implementation should be risk-centered, with mandatory red-flag symptom warnings and explicit care-seeking thresholds delivered in a structured format. Future studies should incorporate multi-turn dialogues, repeated assessments across multiple time points, and fine-grained safety endpoints for further validation.

## Data Availability

The original contributions presented in the study are included in the article/Supplementary Material, further inquiries can be directed to the corresponding authors.
